# Diabetes and other endocrine-metabolic abnormalities in the long-term follow-up of pancreas transplantation

**DOI:** 10.1186/s40842-016-0032-x

**Published:** 2016-07-15

**Authors:** Marcio W Lauria, Antonio Ribeiro-Oliveira

**Affiliations:** Department of Internal Medicine (Endocrinology section and Transplantation unit), Federal University of Minas Gerais, Rua Alfredo Balena, 190, 30130-100 Belo Horizonte, MG Brazil

**Keywords:** Pancreas transplantation, Diabetes, Hyperlipidemia, Bone loss

## Abstract

Pancreas transplantation (PTX) has been demonstrated to restore long-term glucose homeostasis beyond what can be achieved by intensive insulin therapy or islet transplants. Moreover, PTX has been shown to decrease the progression of the chronic complications of diabetes. However, PTX patients require chronic use of immunosuppressive drugs with potential side effects. The long-term follow-up of PTX patients demands special care regarding metabolic deviations, infectious complications, and chronic rejection. Diabetes and other endocrine metabolic abnormalities following transplantation are common and can increase morbidity and mortality. Previous recipient-related and donor-related factors, as well as other aspects inherent to the transplant, act together in the pathogenesis of those abnormalities. Early recognition of these disturbances is the key to timely treatment; however, adequate tools to achieve this goal are often lacking. In a way, the type of PTX procedure, whether simultaneous pancreas kidney or not, seems to differentially influence the evolution of endocrine and metabolic abnormalities. Further studies are needed to define the best approach for PTX patients. This review will focus on the most common endocrine metabolic disorders seen in the long-term management of PTX: diabetes mellitus, hyperlipidemia, and bone loss. The authors here cover each one of these endocrine topics by showing the evaluation as well as proper management in the follow-up after PTX.

## Background

During the past decades, pancreas transplantation (PTX) has evolved into a procedure mainly reserved for type 1 diabetes patients undergoing simultaneously kidney transplantation, although it has also been performed as an isolate procedure [1]. Importantly, it has significantly improved diabetes related quality of life as well as life expectancy when compared to kidney only recipients [2]. However, there is a paucity of publications as related to the endocrine follow-up evaluation and management to this population of diabetic patients after pancreas transplantation.

A Pubmed search was conducted searching for terms “pancreas transplantation AND metabolism”, “pancreas transplantation AND diabetes”, “pancreas transplantation AND hyperlipidemia”, “pancreas transplantation AND bone disease”. We have included only English written articles, and we have tried to prioritize prospective studies. However, due to the lack of available data concerning pancreas transplantation and metabolic abnormalities, we have also included retrospective, transversal and case reports studies.

## Main text

PTX is the implantation of a healthy pancreas (usually from a deceased donor) into a patient who typically has type 1 diabetes. More than 35,000 PTXs have been reported worldwide [3]. Eighty-four percent of PTX procedures are performed along with kidney transplantation (both organs coming from the same donor) in diabetic patients with renal failure. This is referred to as simultaneous pancreas-kidney (SPK) transplantation. Nine percent of PTXs are performed after a previous successful kidney transplantation, which is termed pancreas-after-kidney transplantation (PAK). The remaining 7 % of cases are performed as pancreas transplantation alone (PTA) in nonuremic patients with very labile difficult to manage diabetes.

The number of US PTX has declined by over 20 %, while the overall number of pancreas transplants performed outside the US has increased since 2010. The decline in US numbers is predominantly due to the decline in PTA and PAK. With the decline in the number of transplants, a change towards better pancreas donor selection has been observed [3]. Furthermore, the number of PTX in patients with type 2 diabetes and end-stage renal disease has increased, and accounted for 9 % of all SPK recipients in 2010–14 [3].

Pancreas transplantation is superior to intensive insulin therapy with respect to  glycated hemoglobin (A1C) normalization and shows the additional physiological property of proinsulin and C-peptide release [4]. With new advances in immunosuppression and changes in surgical techniques, patient survival and pancreas graft function have been improving, with PTX being widely employed as a treatment modality for patients with diabetes,especially those with established nephropathy [1, 3]. Nevertheless, PTX remains a complex procedure, which is still associated with high general surgical morbidity. In addition, graft failure, side effects of immunosuppressive agents, opportunistic infections, and cardio- and cerebrovascular problems can increase morbidity and mortality following transplantation [1, 5, 6].

Diabetes and other metabolic abnormalities have frequently been observed after PTX, which can influence its long-term outcomes. These disorders have been related to various factors such as immunosuppressive drug side effects, chronic rejection, and recipient lifestyle after transplantation. Early recognition of these abnormalities can provide for more opportune treatment [1, 4, 5].

This review will focus on the most common endocrine and metabolic disorders related to PTX, such as diabetes, hyperlipidemia, and bone loss. It is noteworthy to mention that due to the absence of clinical guidelines developed through the GRADE approach to this population, our suggested evaluation and follow-up may eventually show variations from other centers, although we have tried to summarize them through the best available sources.

## Diabetes after pancreas transplantation

### Glucose metabolism disorders

No other insulin delivery regimen can achieve the level of physiologic glycemic regulation than that obtained with PTX. It has proved more effective in lowering A1C than intensive insulin treatment or islet transplants [1, 5–7]. Restoration of β‑cell secretory capacity, improvement in glucose counter-regulation, and return to hypoglycemia awareness can all be achieved with a successful PTX [8]. Normalization of A1C occurs within weeks to months and can last for more than 15 years [9]. Transient hyperglycemia may occur within the first six months due to acute or chronic rejection, pancreatitis, or a marked increase in insulin resistance due to weight gain. Immunosuppressant medication effects [5, 6], such as steroids, calcineurin inhibitors (tacrolimus, in particular), sirolimus, and mycophenolate have also been linked to posttransplantation hyperglycemia [10].

Hypoglycemia may also occur following PTX [11, 12]; however, severe episodes (with or without symptom awareness) are rare. By 3 months after PTX, glucagon secretion and hepatic glucose production in response to hypoglycemia also return to normal [13]. Although epinephrine and growth hormone responses to hypoglycemia improve after pancreas transplant, these do not return to normal [14].

Hyperinsulinism is frequently observed after PTX, as are elevated C-peptide and proinsulin concentrations. One likely explanation for these abnormalities—among other reasons, such as the side effects of immunosuppression—is the systemic release of insulin via the iliac vein, as there is no first-pass effect of insulin through the liver [15]. It has been reported that drainage of the venous effluent of the pancreas transplant via the superior mesenteric or portal vein (portal venous drainage) allows comparable blood glucose control but lower insulin levels, as well as possible advantages in metabolic control over systemic venous drainage. However, this mechanism has not been accepted by all authors [16, 17]. Interestingly, a recent report [18] in SPK patients at 1 and 5 years posttransplantation showed that peripheral insulin resistance with homeostatic model assessment (HOMA-IR) >4 was related to decreased pancreatic graft survival. However, data from HOMA-IR in transplanted patients is lacking and needs to be interpreted cautiously.

The prevalence of diabetes after pancreas transplantation is variable, depending on the frequency of assessment, duration of follow-up, and, most importantly, the case definition for diabetes or glucose intolerance. The estimates of this prevalence is rather imprecise as some have taken insulin use as the definition for diabetes while others excluded technical failures or acute rejection to this assessment.

Hyperglycemia after PTX may have more than one of the following explanations: graft failure (due to rejection, thrombosis, or pancreatitis), new-onset diabetes (type 2 or secondary to corticosteroids or immunosuppressive drugs), immunosuppressant-induced islet cell toxicity (particularly tacrolimus in high doses), or recurrent autoimmune type 1 diabetes [19].

Interestingly, the recurrence of type 1 diabetes has been related to the presence of the autoantibodies to the zinc transporter 8 and cannot be explained by genetically encoded amino acid sequence donor-recipient mismatches for this autoantigen [20].

Most cases of late hyperglycemia are attributed to chronic rejection, which, along with technical failure, is the most common cause of hyperglycemia following PTX. Chronic rejection accounts for approximately 50 % (or more) of the grafts that survive over five years [21]. Pancreas failure in the long term can be related to the donor, to the surgical manipulation of the graft, or to the recipient (Table 1).

**Table 1:** Related risk factors for diabetes mellitus following pancreas transplantation

• Donor: older age and high body mass index • Surgical procedure: extensive manipulation of the graft and long ischemic period • Recipient: higher body mass index and posttransplant weight gain; donor age, hepatitis C virus and donor-positive with recipient-negative CMV status • Type of pancreas transplantation: pancreas transplantation alone and pancreas after kidney transplantation • Immunosuppressive regimen: prolonged use of glucocorticoids and use of tacrolimus • Follow-up: number of acute rejection episodes, loss of the kidney graft in pancreas-kidney transplantation, and long duration of the transplant	

Neidlinger et al. [22] analyzed 674 PTX recipients over a 10-year period, with mean follow-up of more than 6 years. The incidence of posttransplant diabetes mellitus (PTDM) was 14 % and 25 % at 3 and 10 years after PTX, respectively. Higher recipient body mass index and posttransplant weight gain, donor age, and donor-positive with recipient-negative CMV status were associated with PTDM after controlling for possible confounding factors [22]. Dean et al. also found a relationship between PTDM, high pretransplant insulin requirements and episodes of acute rejection [6]. Hilling et al. demonstrated that recipient factors are more important in explaining differences in pancreas graft survival than donor factors [23]. Interestingly, the incidence of post-transplant diabetes after successful PTX is lower than that for other solid organ transplants despite the use of the same immunosuppressive drugs [24].

Both PAK and PTA entail higher incidence of graft loss from chronic rejection compared with SPK [3]. For the PAK and PTA categories, such risk remains high even after 1 year posttransplantation, thus requiring greater doses of immunosuppressive drugs and increasing toxicity risks [25]. In one study developed by our group comparing long-term follow-up between PTA and SPK patients, we showed that PTA patients exhibited higher tacrolimus levels and worse renal function [26].

### Evaluation

Fasting glucose and A1C should be ordered at all consultations. If A1C is elevated or fasting glucose exceeds 100 mg/dl, unassociated with a recent rejection episode, an oral glucose tolerance test (OGTT) should be performed [27].

There are no specific criteria to diagnose diabetes after pancreas transplantation and so it is recommended to adopt the Second International Consensus of Post -Transplant Diabetes Mellitus [28]. The threshold to define diabetes is based on data from non-transplant patients: fasting glucose ≥ 126 mg/dl on more than one occasion, random glucose ≥200 mg/dl with symptoms or a 2-h glucose level after a 75-g oral glucose tolerance test ≥200 mg/dl [27]. Although A1C ≥ 6.5 % can be used to diagnose diabetes in the general population, it should not be used alone in post-transplant diabetes mellitus, particularly in the first year after transplantation [28].

Pancreas transplantation recipients presenting with recurrent hyperglycemia should have their C-peptide levels measured and undergo pancreas biopsy to distinguish between rejection, recurrence of type 1 diabetes, and onset of type 2 diabetes [4]. Duplex sonographic scanning of the allograft could be helpful in excluding thrombosis and rejection. However, sonography is less accurate than biopsy to diagnose rejection [29, 30]. Ultrasound-guided pancreas biopsy is otherwise considered the gold standard for the diagnosis of an allograft dysfunction.

Unfortunately, hyperglycemia is a late marker of chronic graft rejection. Although plasma glucose has high specificity (90 % to 95 %), it is the least sensitive marker of rejection [31]. Hence, identification of the subjects at risk of returning to the diabetic state due to graft loss or any other cause is difficult and often delayed. Consequently, appropriate treatment becomes unfeasible.

Several tests have been proposed to identify patients at high risk for all-cause pancreatic graft failure. Slight alterations in glucose metabolism seem to appear earlier and might be predictive of pancreatic graft failure. Pfeffer et al. performed OGTT 1.7 years after SPK transplantation on average and showed that impaired glucose tolerance predicted risk of graft failure in 10 years [32]. Battezzati et al., taking blood samples at 2 h intervals for plasma glucose, serum insulin, and C-peptide levels, reported that mean 24-h glucose greater than 127 mg/dL at 1 year posttransplantation was the best predictive index of return to a diabetic state [33]. The intravenous glucose tolerance test and the arginine-induced insulin secretion test have also been used as rejection markers [34, 35]. In islet transplantation patients, glucose variability and a higher frequency of glucose levels above 140 mg/dL determined by the continuous glucose monitoring system (CGMS) have proved useful early indicators of graft dysfunction [36]. We have also demonstrated that 72 h mean glucose measured by CGMS in PTX patients with normal oral glucose tolerance was associated with chronic rejection in a 5 year follow-up [37]. Recently, Hiratsuka et al. have described the utility of peripheral plasma fasting serum C-peptide response to 1 mg of glucagon intravenously to predict insulin-free treatment [38]. However, further studies are warranted, since biochemical parameters and imaging tools still lack diagnostic accuracy to detect early graft failure, especially for PTA and PAK.

In SPK recipients, serum creatinine levels have been used to diagnose rejection, since kidney graft rejection usually precedes or is concurrent with rejection of the pancreas graft [19].

Urinary amylase levels can be monitored in pancreas recipients with bladder-drained exocrine secretions [39, 40]. An analysis result of a 12-h or 24-h urine collection in which amylase levels have declined 50 % or more from baseline suggests rejection or pancreatitis. Serum amylase and lipase levels provide additional means for following pancreas function, especially in the case of enterically drained grafts. However, they lack the sensitivity and specificity of urinary amylase measures [4]. Long-term graft survival is greater for SPK vs PAK or PTA transplants, which partly explains the decrease in the number of these two last transplant modalities in the past decade [41]. Indeed, in SPK the pancreas and kidney are usually obtained from the same deceased donor and, therefore, changes in kidney function can be used to monitor whether rejection is occurring at either organ.

Table 2 summarizes the recommended tests for identifying patients at risk for pancreatic graft dysfunction.

**Table 2:** Tests for identifying pancreas graft dysfunction

• Urinary amylase levels (only for bladder- drained transplants) • Serum amylase and lipase levels • Fasting glucose levels (consider OGTT if >100 mg/dl) • A1C • Creatinine (in SPK patients) • Duplex sonographic scanning of the allograft (when thrombosis and/or rejection are suspected) • Ultrasound- guided biopsy (gold standard for rejection)	

### Management

Strict glycemic control is the cornerstone of therapy, which can preserve residual β-cell function and eventually recover it when glucotoxicity is corrected. Blood glucose levels should be less than 110 mg/dL before meals and less than 180 mg/dL postprandial [42].

Insulin therapy is generally preferred because of its superior results in controlling diabetes, in addition to being more predictable and rapidly titrated. Insulin therapy is mandatory in the setting of ketoacidosis or metabolic decompensation with unintended weight loss. Furthermore, insulin should be used when hyperglycemia derives from rejection or other causes of graft failure. Further, insulin therapy is mandatory when hyperglycemia derives from rejection or other causes of graft failure. Despite extensive clinical experience with a variety of insulin types, no prospective studies have addressed the relative efficacy of specific insulin regimens for treating posttransplant diabetes [42].

When the cause of hyperglycemia is new-onset type 2 diabetes, oral agents can be used, although this has not been clearly defined for PTX. They should be attempted in patients with mild hyperglycemia with preserved/robust c-peptide. Metformin improves insulin sensitivity, most notably in the liver. Given the risk of metformin-induced lactic acidosis, this agent is contraindicated for patients with severe renal or hepatic dysfunction. Sulfonylureas stimulate insulin secretion and can be an option. However, these drugs are associated with an increased risk of hypoglycemia and can also contribute to β-cell exhaustion. Thiazolidinediones, which primarily target insulin resistance at the level of skeletal muscles and adipocytes, have been evaluated as a potential therapeutic option and seem to be a safe and effective treatment. Special attention must be given to the side effects of thiazolidinediones, such as weight gain, hepatotoxicity, edema, cardiac failure, and increased fracture risk. Agonists of GLP-1 and oral dipeptidyl peptidase inhibitors have not been consistently investigated and thus cannot be recommended, although they might have a positive effect by stimulating insulin secretion in transplant recipients [43].

Although current clinical evidence is largely anecdotal, tailoring of immunosuppression should be considered in patients with poorly controlled diabetes despite therapeutic lifestyle changes and pharmacologic interventions. In this context, therapeutic options include reduction in corticosteroid and/or calcineurin inhibitors dose, and conversion from tacrolimus to cyclosporine or sirolimus [44, 45].

Steroid avoidance or withdrawal for PTX patients has been a matter of debate. A recent review by Montero et al. [46] concluded that the available data, including randomized controlled trials, are still insufficient to firmly infer on the harms and benefits of steroid withdrawal in pancreas transplantation. A study by Amodu et al. [47] showed that steroid maintenance is not associated with the risk of death or graft failure although increasing the risk of infectious complications.

## Hyperlipidemia after pancreas transplantation

### Lipid metabolism disorders

Hyperlipidemia after solid organ transplantation occurs in 60 % to 80 % of recipients of immunosuppressive therapy [48]. High triglycerides and low density lipoprotein cholesterol (LDL-c) levels are two of the most important lipid disorders found in those patients. Combined hyperlipidemia is also common. Many risk factors can contribute to the development of dyslipidemia after PTX, such as older age, obesity, posttransplantation weight gain, pretransplantation hyperlipidemia, male gender, graft dysfunction, proteinuria, new-onset diabetes, prednisone dosage, and the type of immunosuppression regimen (Table 3).

**Table 3:** Risk factors contributing to the development of dyslipidemia following pancreas transplantation

• Older age • Obesity/Overweight • Weight gain after transplantation • Pretransplantation hyperlipidemia • Male gender • Graft dysfunction • Proteinuria • New-onset diabetes • Prednisone dosage • Immunosuppressive agents: sirolimus (particularly), tacrolimus, cyclosporine	

Sirolimus has been found to be associated with greater increases in triglycerides and cholesterol, although tacrolimus and cyclosporine also have deleterious effects on lipid metabolism [5, 10].

The majority of PTX patients have preexisting dyslipidemia, since they are generally poorly controlled diabetes patients, most of them with advanced nephropathy. Compared to preoperative levels, patients who underwent SPK experience improvements in their lipid profile, while those submitted to PTA remain with stable lipid levels [49]. Patients who underwent PTA exhibit similar lipid levels to SPK patients posttransplantation, which suggests that the contrasting clinical course seen between these two groups is attributable to preoperative lipid profile differences [26]. Relative to PTA patients, SPK patients have significantly lower levels of LDL-c and use significantly fewer lipid-lowering medications [50]. Portal venous instead of systemic venous drainage of the pancreas allograft does not seem to impact lipid metabolism in the long-term follow-up [17]. Steroid withdrawal regimens have been related to lower prevalence of hyperlipidemia after PTX [51].

Qualitatively, a persisting profile of potentially atherogenic alterations in lipoprotein lipase, cholesteryl ester transfer, and lipoprotein composition has been reported after PTX [52]. However, the findings and interpretation of those data have been questioned by others [53].

### Evaluation

In the first 3 months following PTX, lipid levels are quite unstable because of high doses of immunosuppressive drugs [54]. After that, levels usually stabilize during the first year posttransplantation. It is important to avoid misleading situations when interpreting the results, such as acute stress conditions and secondary causes of dyslipidemia.

Monitoring lipid profile at least every 6 months following transplantation is advisable.

### Management

Due to the high incidence of atherosclerotic disease events and the absence of specific studies, PTX patients should be considered high risk and treated to maintain LDL-c <100 mg/dl, according to published guidelines for kidney transplant patients [55].

A diet-oriented approach should be the first line treatment in hyperlipidemia [56]; changes in the immunosuppression regimen should also be considered. Tacrolimus appears to have fewer adverse effects on lipids than cyclosporine, which is not explained by concomitant steroid administration. Cross-over studies with renal transplant patients have shown improvement in lipid profile after conversion from cyclosporine to tacrolimus [57, 58].

Statins are considered the cornerstone of drug therapy in posttransplantation hypercholesterolemia. The outcomes and benefits of statins based on heart and kidney clinical studies provide a solid rationale to support their use in organ transplantation. Furthermore, statins can potentially exert cholesterol-independent immunosuppressive effects [59]. Use of statins in PTA patients may lead to improved outcomes. Whether this is due to cardiovascular protection or other factors unrelated to lipid lowering remains unclear [60].

Similarly to kidney transplant, statin treatment has uncertain effects on overall mortality, stroke, kidney function, and toxicity outcomes in PTX recipients. Further studies would improve our knowledge of the benefits and harms of statin treatment regarding cardiovascular events in this clinical setting [61].

Since statins can cause hepatotoxicity, it is important to monitor liver function after statin introduction. Moreover, we should be cautious about the myotoxic effects of statins and the risk of rhabdomyolysis due to interaction with drugs that inhibit the cytochrome P450 isoenzyme and thus increase statin levels [62]. Regarding associations between statins and tacrolimus, animal studies have shown a pronounced interaction, similar to that described for cyclosporine. Not all statins are metabolized by the same enzyme; therefore, they exhibit a different drug interaction and safety profile [63]. Statin-induced dysglycemia is another concern that has recently been described in kidney transplants [64]. The choice of a statin should be based on individual patient requirements and adapted according to treatment response. Pravastatin and fluvastatin have been considered the safest statins to be used in transplanted patients [65]. However, given their low potency, a high-potency statin may be necessary in patients with significant dyslipidemia [66, 67].

Concerning hypertriglyceridemia, fibrates should be used cautiously, since they may induce renal dysfunction. Gemfibrozil seems to be devoid of this side-effect [68]. Interestingly, in the Fenofibrate Intervention and Event Lowering in Diabetes (FIELD), fenofibrate reduced albuminuria and slowed estimated renal function loss over 5 years, despite initially and reversibly increasing plasma creatinine. Confirmatory studies are merited [69].

## Bone diseases following pancreas transplantation

### Calcium and bone metabolism disorders

Bone complications are another major source of late morbidity for PTX patients. Fractures after SPK transplant are common: 45 to 49 % within the first 2 years, which is one of the highest fracture rates reported for any organ transplant [70]. Cortical osteoporosis is prevalent in SPK recipients at the time of transplantation and shows early progression after transplantation [71]. Over the long term, bone densitometry values and fracture risk seem to stabilize or improve, especially in steroid sparing protocols and/or aggressive osteoporosis treatment [71, 72].

The disturbances in bone and mineral metabolism observed after transplantation are largely determined by preexisting factors at the time of transplantation (advanced age, female gender, long duration of pretransplant kidney failure, history of pretransplant fracture) and by factors inherent to the transplant status, such as kidney function and use of immunosuppressive agents [73, 74] (Table 4).

**Table 4:** Factors related to bone and mineral metabolism disturbances following pancreas transplantation

• Preexisting: older age, female gender, long duration of pretransplant kidney failure, history of pretransplant fracture, severity of hyperparathyroidism secondary to kidney disease • Inherent to transplant status: kidney function, use of immunosuppressive agents (especially glucocorticoids and calcineurin inhibitors) • Other factors commonly observed after transplantation: hypogonadism, vitamin D deficiency, persistent parathyroid disease, thyroid abnormalities	

Compared to PTA recipients, kidney transplant recipients have the disadvantage of presenting for transplantation with preexisting osteodystrophy, which is difficult to predict from routine laboratory or radiologic investigations and it may persist, improve, or worsen after transplantation [75]. Interestingly, SPK has been associated with a significant 31 % reduction in fracture risk compared to kidney alone transplantation in men with type 1 diabetes after adjustment for fracture covariates [76]. It is not known whether the apparent benefit of SPK is due to improved bone strength or fewer falls observed with the restoration of euglycemia. Furthermore, a recent study by Rocha et al. [77] showed that, in SPK patients followed for at about 3 years, a gain in BMI was significantly predictive of bone mass improvement whereas an increasing in serum levels of alkaline phosphatase was significantly associated with a decrease in the same parameter.

After kidney transplantation, many laboratory features of chronic kidney disease improve; however, abnormally high or low bone turnover rates have been reported in bone biopsy studies [78, 79].

Corticosteroids exacerbate bone loss by suppressing osteoblastic activity and activating osteoclastic resorption. Calcineurin inhibitors also have direct negative effects on bone [80–82]. Factors such as hypogonadism, vitamin D deficiency, adynamic bone disease, previous or ongoing parathyroid disease, previous uncontrolled diabetes, and thyroid function abnormalities can also contribute to bone loss [19]. Persistently increased PTH concentrations can be found in as many as 75 % of patients at 1 year posttransplantation; this is largely due to failure of the enlarged glands to involute. Elevated FGF23 levels and decreased Klotho expression in the parathyroid gland may play a role in the pathogenesis of hyperparathyroidism after kidney transplantation [83]. A major determinant of persistent posttransplant hyperparathyroidism is its severity at the time of transplantation, corresponding to pretransplantation PTH levels [84, 85]. Persistent hyperparathyroidism is a major risk factor for fractures after kidney transplantation [86]. The foot and ankle are the most common fracture sites after PTX, accounting for over 50 % of the cases. This suggests that preexisting diabetes-related deformities could be an etiologic factor. Charcot neuroarthropathy has been diagnosed in 4.6 % of SPK recipients during the first year posttransplantation [87, 88]. In addition, a variety of other anatomic sites are affected [89].

### Evaluation

No clinical trial data to inform clinical recommendations for bone disease in PTX patients are available. Most data is limited to before-after data or registry studies. In view of this, guidance for renal transplantation patients is taken as a reference, although it needs to be applied cautiously [55].

Close monitoring of serum calcium, phosphate, 25-hydroxy-vitamin D (25 OH-D), and PTH concentrations are recommended for SPK and PAK recipients [19]. Bone mineral density should be measured in the first 3 months after transplantation, when patients have a calculated clearance of creatinine above 30 mL/min, and then repeated on a regular basis [55]. The work-up should also include periodical determination of thyroid and sex hormone status in eligible populations [4]. Biomarkers of bone turnover could be useful in individualizing therapy to prevent or treat bone loss after transplantation [90]. For PTA recipients, evaluation should be done considering the presence of other fracture risk factors such as peripheral neuropathy, poor muscle strength, balance impairment, visual acuity reduction, and propensity to falls.

### Management

Vitamin D deficiency and insufficiency should be corrected by using treatment strategies recommended for the general population to achieve a serum 25 OH-D concentration of at least 20 ng/mL to prevent and treat osteometabolic diseases [55]. Low 25 OH-D has been independently associated with an increased risk of all-cause mortality in kidney transplantation [91]. Pleotropic effects of vitamin D, such as immune response regulation, and protective effects from cardiovascular disease, cancer, and infections seem to be very attractive to the transplanted population. However, more solid data are needed to confirm this and to set the optimal level of serum Vitamin D supplementation in order to attain the best clinical outcome [92]. Calcium supplementation (500 mg elemental calcium daily) appears to lower bone resorption after transplantation and should be routinely prescribed—especially in regimens with high doses of immunosuppressive drugs [93]. Calcium dietary intake of at least 1 g daily should also be advised.

In patients with established osteoporosis (presence of densitometric osteoporosis or fractures associated with osteopenia), anti-resorption therapy, usually with bisphosphonates orally or intravenously, is warranted [90, 94]. Bisphosphonates may also be beneficial to prevent bone loss after transplant, although this needs to be more clearly established.

In patients with persistent hyperparathyroidism after transplantation, calcitriol may provide additional benefits in reducing bone loss [94]. A calcium sensor blocker (Cinacalcet) has not yet been approved for use in transplanted patients and evidence of its safety in this clinical setting is still incomplete. In refractory and persistent cases of hyperparathyroidism, parathyroidectomy should be considered [95].

### Other endocrine and metabolic diseases

Thyroid diseases are common in the general population and, therefore, can be often observed in the pancreas transplanted population. Furthermore, patients with type 1 diabetes show increased incidence of autoimmune thyroid diseases. However, no relationship between pancreas transplantation and thyroid disesases has been described. Thus, screening and management of thyroid diseases in pancreas transplanted patients is not different from the general population.

Hypogonadism in men and women have been reported before and after pancreas transplantation, mainly in those patients with SPK and probably due to the effects of the end stage renal disease in the hypothalamic-gonadotroph axis function. Immunosupressive drugs may play a role in these disorders. However, in the majority of the cases, gonadal function actually recovers after transplantation [96]. Glucocorticoid metabolism alterations may occur in transplanted patients and are related to its chronic use to prevent organ rejection. These patients should be advised to increase or reassume glucocorticoid therapy during stress to avoid adrenal insufficiency.

## Conclusions

Disturbances in the metabolism of carbohydrates, lipids, and bone mineral are prevalent problems observed in the follow-up of PTX patients. In addition to causing morbidity, such disturbances can indirectly increase the risk of death associated with cardiovascular diseases and fractures. Previous recipient-related and donor-related factors and others inherent to the transplant act together in the pathogenesis of these abnormalities. Early recognition of such disturbances is the key to timely treatment; however, adequate tools to achieve this goal are frequently lacking. Further studies are needed to define the best approach for PTX patients.

PTX is a technically demanding procedure which is associated with the above-mentioned abnormalities in the endocrine system beyond infectious and rejection complications. Furthermore, patient follow-up is complex due to multiple interfering factors, where immunosuppressive drugs play an important role. Therefore, despite the good outcomes recently achieved in centers with a specialized transplantation unit as well as recent advances to the field, PTX should still be considered the last resort treatment in patients whose diabetic complications have become life-threatening or enough burdensome to be maintained with the current available diabetes treatment.

## Abbreviations

PTX, pancreas transplantation; SPK, simultaneous pancreas kidney transplantation; PAK, pancreas after kidney transplantation; PTA, pancreas transplantation alone; A1C, glycated hemoglobin; HOMA-IR, homeostatic model assessment; PTDM, posttransplant diabetes mellitus; OGTT, oral glucose tolerance test; CGMS, continuous glucose monitoring system; LDL-c, low density lipoprotein cholesterol; 25 OH-D, 25hydroxy vitamin D
